# Preoperative Denosumab may increase the Risk of Local Recurrence of Giant-cell Tumor of Bone Treated with Curettage: A Systematic Review and Meta-analysis

**DOI:** 10.7150/jca.50575

**Published:** 2021-01-01

**Authors:** Yongzhao Zhao, Zhenyu Cai, Xiaodong Tang, Zhiye Du, Yi Yang, Wei Guo

**Affiliations:** Musculoskeletal Tumor Center, Peking University People's Hospital, No. 11 Xizhimen South Street, Xicheng District, Beijing 100044, China.

**Keywords:** Denosumab, Giant-cell Tumor of Bone, Local recurrence, Meta-analysis

## Abstract

**Objective:** This systematic review and meta-analysis aimed to determine the effect of preoperative denosumab on the local recurrence of giant-cell tumor of bone (GCTB) treated with curettage.

**Methods:** PubMed, Embase, Cochrane Library, and Web of Science were comprehensively searched. The following data were analyzed using meta-analysis: local recurrence rate of patients receiving denosumab followed by curettage (denosumab group), local recurrence rate of patients receiving curettage only (control group), and a comparison of the local recurrence rates of the two groups.

**Results:** Nine studies that contained 672 patients with GCTB were included in this review. Patients in the denosumab group (preoperative denosumab followed by curettage) had a higher risk of local recurrence compared with those in the control group (curettage only) (odds ratio = 3.04, 95% confidence interval = 1.48-6.22, *P* < 0.01). The association between preoperative denosumab and local recurrence remained significant in most of the subgroup analyses, except for those with sample sizes < 59 (*P* = 0.09), sacral GCTB (*P* = 0.42), and usage of postoperative denosumab (*P* = 0.38).

**Conclusions:** Preoperative denosumab may increase the risk of local recurrence of GCTB treated with curettage and should be used with caution in the management of GCTB.

## Introduction

Giant-cell tumor of bone (GCTB) is a rare primary benign bone tumor that accounts for approximately 5% of all primary bone tumors 1. Curettage has become the mainstream therapy for GCTB due to its advantage of preserving the local functional anatomy, such as articular joint surface and nerves. However, the local recurrence rate after curettage remains high despite the usage of local adjuvants (e.g., phenol, peroxide, and liquid nitrogen) 2.

GCTB consists of osteoclast-like giant cells that express the receptor activator of nuclear factor-kappa β (RANK) and neoplastic stromal cells that express the RANK ligand (RANKL); RANKL is an indispensable part in the pathogenesis of GCTB 3. As a full human monoclonal antibody inhibiting RANKL, denosumab has been approved for treating unresectable GCTB or the surgical resection of GCTB that may cause severe morbidity 4, 5. Previous studies indicated that preoperative denosumab could result in beneficial surgical downstaging in the treatment of GCTB 6. Data from other studies suggested that preoperative denosumab might increase the risk of local recurrence after the curettage of GCTB 7-15. *Scoccianti et al.* 13 evaluated the local recurrence rate of GCTB treated with curettage and cryotherapy, and the authors observed a higher local recurrence rate in preoperative denosumab plus the curettage group compared with the curettage-only group (5/12, 41.67% versus 1/9, 11.11%, *P* < 0.05). Similarly, *Errani et al*. 10 observed an increased local recurrence rate in GCTB treated with preoperative denosumab followed by curettage when compared with the curettage-only group (15/25, 60% versus 36/222, 16%, *P* < 0.05). However, *Chen et al.* 8 indicated a comparable local recurrence rate between preoperative denosumab followed by curettage and curettage-only group in sacral GCTB (3/11, 27.27% versus 3/10, 30.00%). A definite conclusion has yet to be obtained about preoperative denosumab on the local recurrence of GCTB treated with curettage because of contradictory results across published studies 7-15. The current study is a systematic review and meta-analysis that investigates the effect of preoperative denosumab on the local recurrence of GCTB treated with curettage.

## Methods

This study was performed according to Preferred Reporting Items for Systematic Reviews and Meta-Analyses 16, and the protocol of this study was registered in PROSPERO (https://www.crd.york.ac.uk/prospero/) (ID: CRD42020167641).

### Eligibility criteria

The included studies should meet the following inclusion criteria: participants (patients with GCTB), intervention (preoperative denosumab followed by curettage), control (curettage only), outcome (local recurrence), and study design (retrospective or prospective studies). The following studies were excluded: case reports, reviews, animal or cell experiments, inefficient data, non-English language, or duplicated patients.

### Information source, literature search, and study selection

PubMed, Embase, Cochrane Library, and Web of Science were comprehensively searched online on February 6, 2020. The following terms were used in the literature search: (“Giant Cell Tumor” OR “Giant Cell Tumor of Bone” OR “Osteoclastoma”) combined with (“AMG162” OR “Denosumab” OR “Xgeva” OR “Prolia”). The details are listed in **Supplementary Table S1**. The study selection was independently conducted by two investigators according to the eligibility criteria, and any disagreement was resolved through group discussion.

### Data collection and summary

In each study, we extracted the following items: name of the first author, publication year, country, institution, study design, sample size, number of total patients in the denosumab group (preoperative denosumab followed by curettage) or control group (curettage only), local recurrence in denosumab group or control group, tumor site, Campanacci stage 3, previous surgery (primary or recurrent cases), usage of chemical adjuncts during the curettage process, duration of preoperative denosumab, usage of postoperative denosumab, follow-up time, and matched or unmatched factors between the two groups. The duration of preoperative denosumab was transformed from dosage of denosumab if only the dosage was reported in specific studies. Data collection was conducted by two investigators independently, and any disagreement was resolved through group discussion.

### Risk of bias in individual studies

The Newcastle-Ottawa scale (NOS), which has been widely used in meta-analyses 17, 18, was applied to evaluate the risk of bias in the included studies 19. NOS contains three main categories, namely, selection, scored with four stars; comparability, scored with two stars; and ascertainment of the outcome, scored with three stars. Any study with a score of 1 in the selection or outcome ascertainment, 0 in any of the three domains, or a total score of less than 5 was deemed to have a high risk of bias 17, 18.

### Statistical analysis

This study used Review Manager 5.3 software (Cochrane Collaboration, London, UK) and Stata 12.0 (StataCorp, College Station, TX) in the meta-analysis. The association between preoperative denosumab and local recurrence was evaluated using odds ratio (OR) with a corresponding 95% confidence interval (CI); a P value less than 0.05 indicated a significant association. The local recurrence rate in the denosumab or control group was determined by pooling the data from the included studies. Heterogeneity among the studies was analyzed using the chi-squared test. A fixed-effects model was used in the absence of significant heterogeneity (*P* > 0.10, I^2^ < 50%); otherwise, a random-effects model was used (*P* < 0.10, I^2^ ≥ 50%). Subgroup analysis was performed to explore the source of heterogeneity. Sensitivity analysis was carried out to assess the influence of individual studies on the overall results of the association between preoperative denosumab and local recurrence in GCTB treated with curettage. Publication bias across the included studies was evaluated using Egger's test and Begg's test by using Stata 12.0; a *P* value less than 0.05 indicated a large publication bias.

## Results

### Study selection

**Figure 1:**
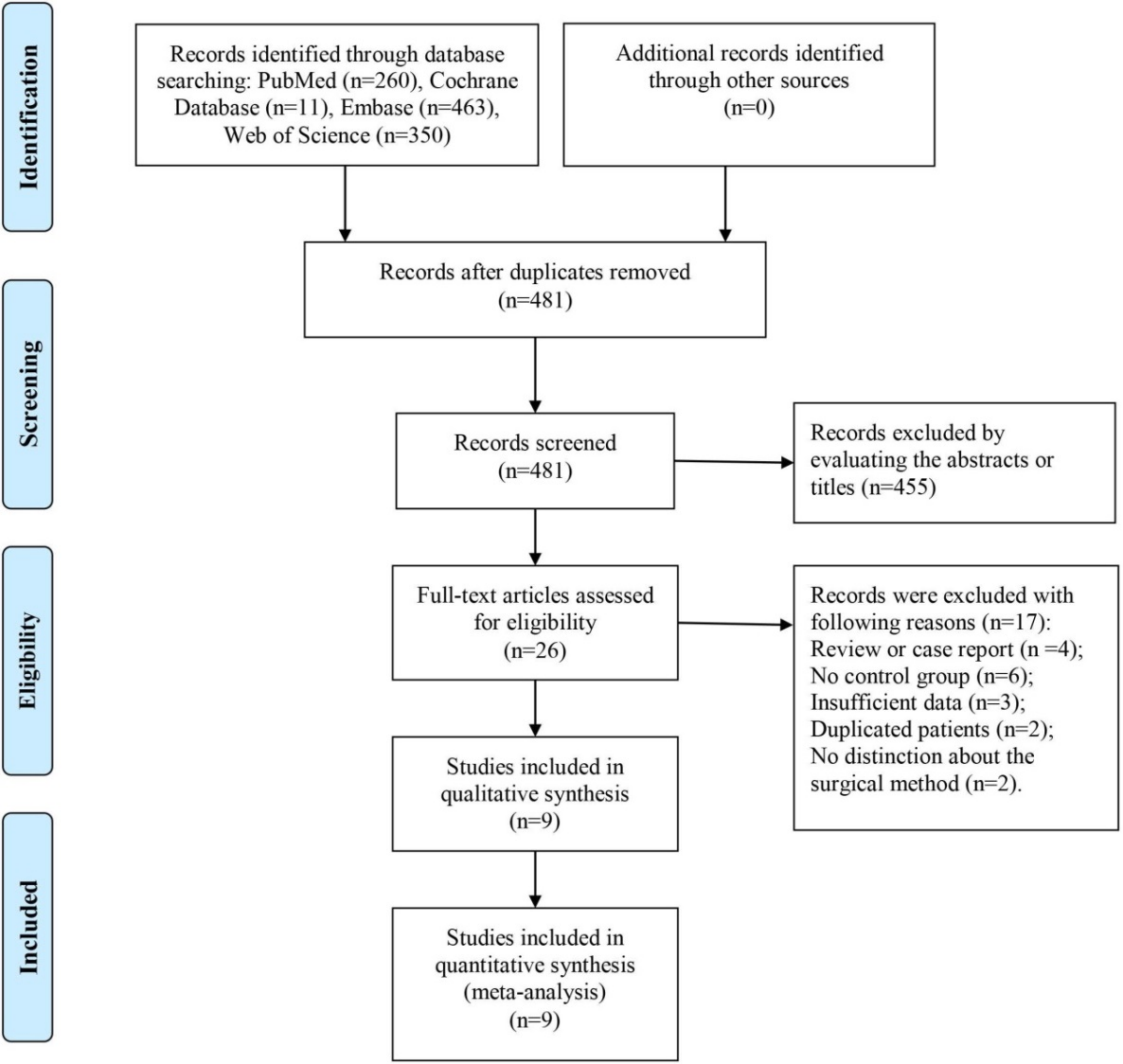
Flow chart of the study selection.

As shown in **Figure 1**, 1,183 records were obtained from four common databases, namely, PubMed (n = 260), Cochrane Library (n = 11), Embase (n = 463), and Web of Science (n = 350). Four hundred and eighty-one records remained after the removal of duplications, and 455 records were directly excluded after the titles or abstracts were scanned. For the remaining 26 records, full texts were carefully evaluated and 17 studies were excluded for the following reasons: review or case report (n = 4), no control group (n = 6), insufficient data (n = 3), duplicated patients (n = 2), and no distinction about the surgical method (curettage or en bloc resection) (n = 2). Finally, nine studies were included in this systematic review and meta-analysis 7-15.

### Characteristics of included studies

The characteristics of included studies are listed in **Table 1** and** Table 1A**. Nine retrospective studies containing 672 patients were included in the analysis 7-15. The denosumab group had 172 patients, and the control group had 500 patients. Five studies were performed in Asia 7-9, 14, 15, and the other four studies were performed in Europe 10-13. Seven studies were conducted in a single medical center 7-10, 12, 13, 15, and two studies were conducted in multiple medical centers 11, 14. The sample size varied considerably across the studies from 16 to 247. With respect to the tumor site, five studies focused on GCTB at all bones 7, 9, 11, two studies focused on sacral GCTB 8, 15, and two studies focused on appendicular GCTB 10, 12. All the studies, except *Fedenko et al.* 11, reported information on the Campanacci stage and previous surgery of cases. Chemical adjunct was used during the curettage in four studies 9, 10, 13, 14, including phenol, ethanol, and cryotherapy. The median duration of preoperative denosumab ranged from 2 months to 8.9 months, and the patients received both preoperative and postoperative denosumab in three studies 9, 10, 14. The median follow-up time ranged from 12 months to 85.6 months among the included studies. Six studies reported the matched factors between the two groups, such as age, gender, and Campanacci stage 7-10, 13, 15. Risk of bias in the studies was evaluated using NOS, and results showed that all the studies had a low risk of bias with scores > 5 (**Table 2**).

### Local recurrence

**Figure 2:**
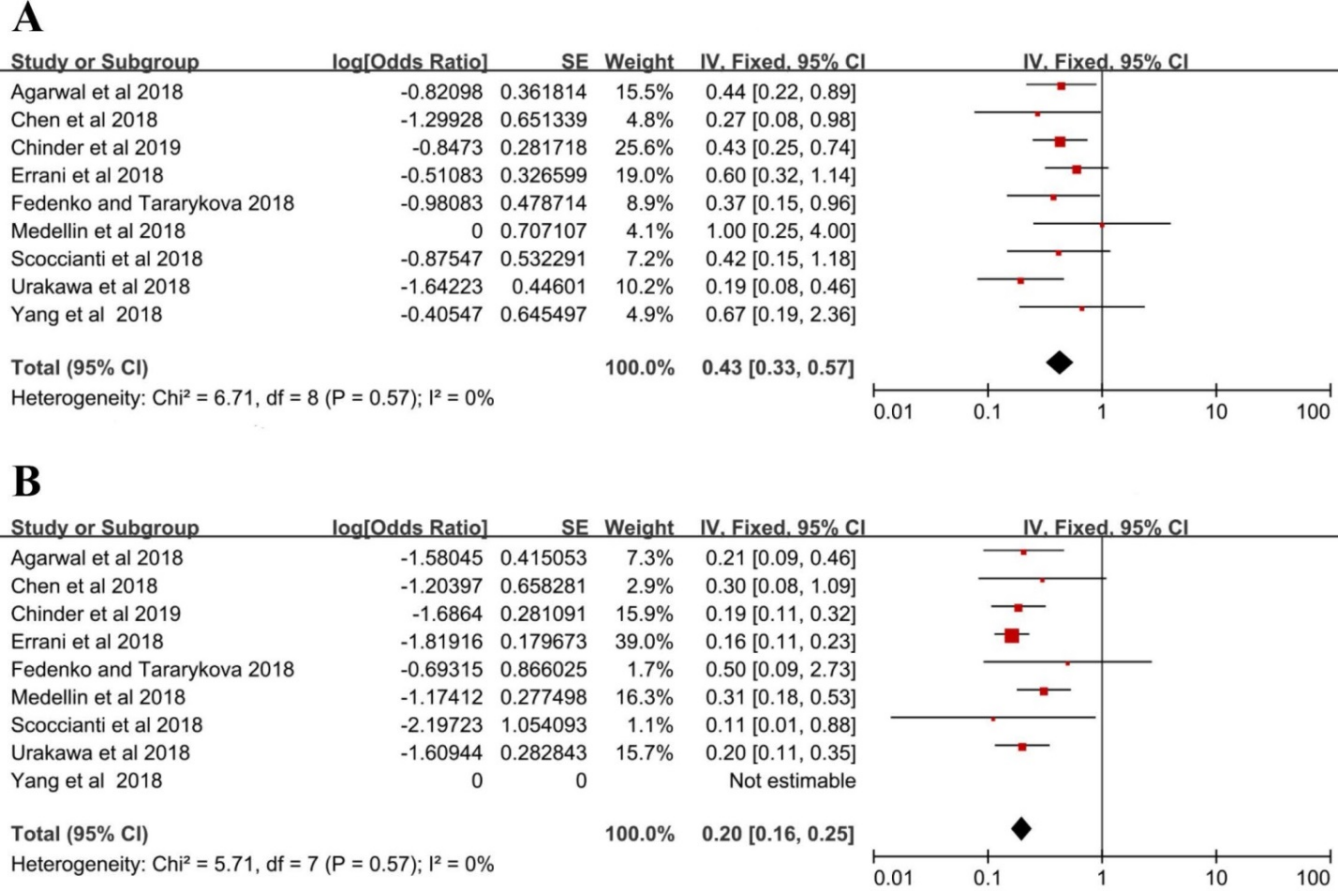
Local recurrence rate (A) denosumab group, (B) control group.

Seventy-two patients suffered from local recurrence in the denosumab group with a local recurrence rate of 43% (95% CI = 33%-57%), and the fixed-effects model was used in studies without heterogeneity (I^2^ = 0%, *P* = 0.57) (**Figure 2A**). In the control group, local recurrence occurred in 96 out of 500 patients, and the local recurrence rate was 20% (16%-25%) with the fixed-effects model (I^2^ = 0%, *P* = 0.57) (**Figure 2B**).

**Figure 3:**
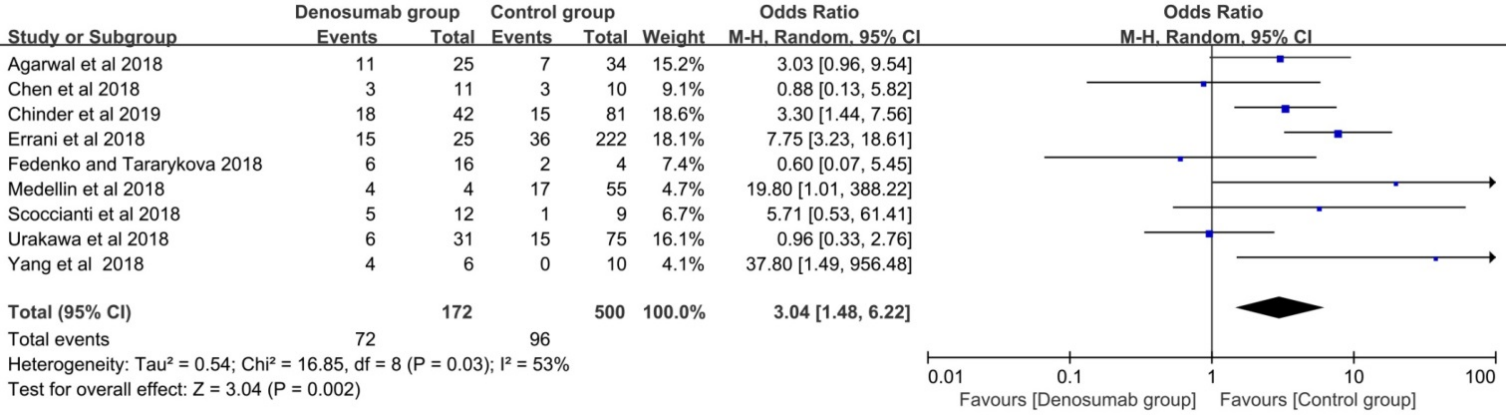
Comparison of local recurrence rate between the denosumab and control groups.

With regard to the comparison of local recurrence between the denosumab and control groups, the random-effects model was used for evident heterogeneity (I^2^ = 53%, *P* = 0.03). Results showed that the patients in the denosumab group had a significantly higher risk of local recurrence compared with those in the control group (OR = 3.04, 95% CI = 1.48-6.22, *P* < 0.01) (**Figure 3**). To explore the source of heterogeneity, subgroup analysis was performed in the following factors: ethnicity, sample size, tumor site, Campanacci stage, whether or not a previous surgery was performed, usage of chemical adjunct, duration of preoperative denosumab, and usage of postoperative denosumab. A higher risk of local recurrence was detected in the denosumab group compared with the control group in most analyses (*P* < 0.05), except for sample sizes < 59 (*P* = 0.09), sacral GCTB (*P* = 0.42), and usage of postoperative denosumab (*P* = 0.38) (**Table 3**).

### Sensitivity analysis

**Figure 4:**
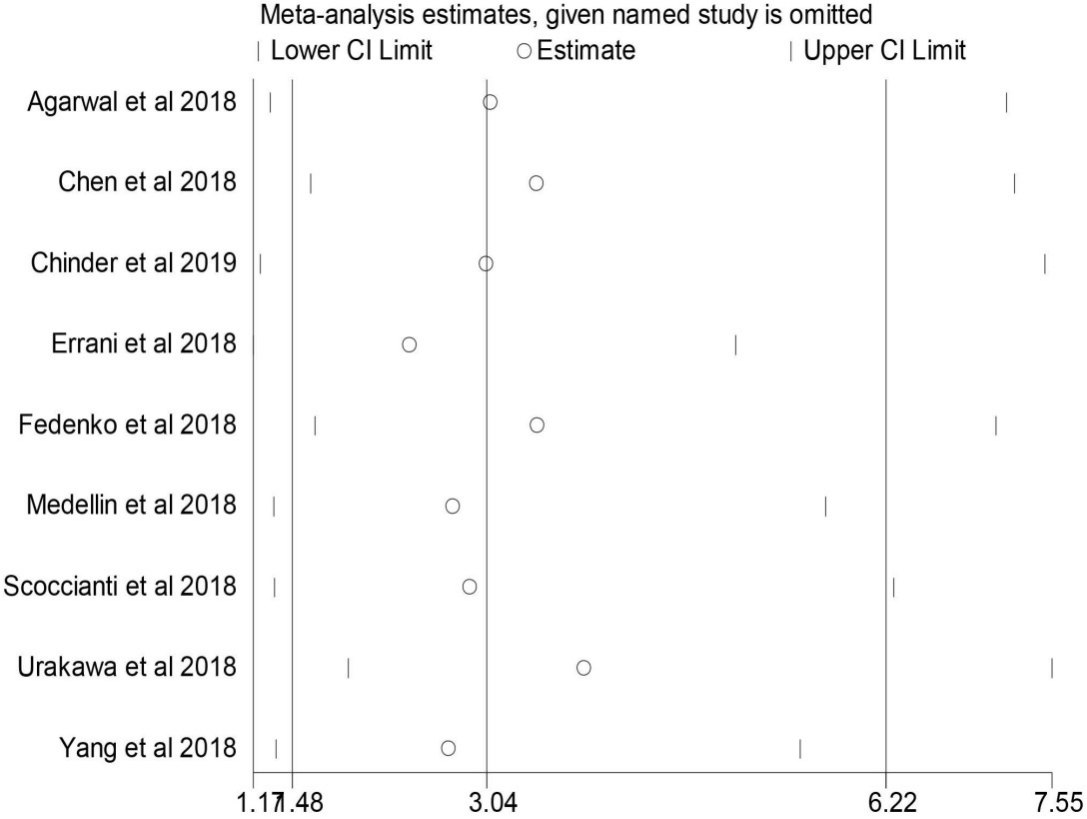
Sensitivity analysis in the comparison of local recurrence rate between the denosumab and control groups.

No individual study dominated the overall results of the association between preoperative denosumab and local recurrence in GCTB treated with curettage, and the removal of any single study did not change the overall conclusion (**Figure 4**).

### Publication bias

**Figure 5:**
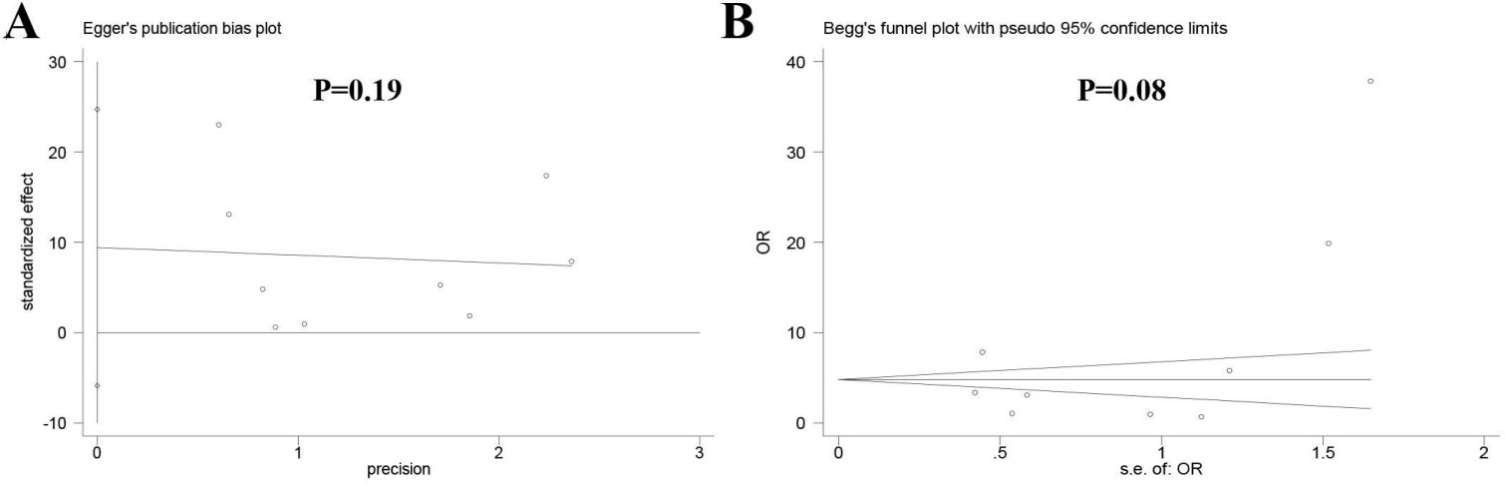
Publication bias among the included studies (A) Egger's test, (B) Begg's test.

No obvious publication bias was observed across the included studies in the meta-analysis of local recurrence between the two groups according to Egger's test (*P* = 0.19) (**Figure 5A**) and Begg's test (*P* = 0.08) (**Figure 5B**).

## Discussion

The use of denosumab prior to surgical therapy has been gradually accepted in the management of GCTB for surgical downstaging, especially in spinal or sacral GCTB or surgery with a probability of causing severe morbidity 5. However, increasing evidence has shown that preoperative denosumab might elevate the risk of local recurrence of GCTB after curettage 7-15. In consideration of the small sample size and contradictory results among published studies 7-15, this systematic review and meta-analysis was performed for the first time to determine the effect of preoperative denosumab on the local recurrence of GCTB treated with curettage.

Several studies have evaluated the local recurrence of GCTB treated with denosumab followed by curettage 20-22. *Niu et al.* 20 retrospectively reviewed 13 patients with GCTB who received denosumab followed by curettage with a median follow-up time of 18.8 (range: 10-31) months; 23.08% (3/13) of patients experienced local recurrence. In *Puri et al.* 21, 44% (11/25) of patients receiving denosumab followed by curettage suffered from local recurrence during a median follow-up time of 34 (range: 24-48) months. *Traub et al.* 22 observed a local recurrence rate of 15% (3/20) in patients treated with denosumab followed by curettage with a median follow-up time of 30 (range: 20-45) months. We integrated the data from nine comparative studies in our analysis and found that 43% of patients receiving preoperative denosumab followed by curettage experienced local recurrence; this value was higher than those in abovementioned studies 20-22. The high local recurrence rate in our research could be explained by the high proportion of spinal or sacral GCTB 8, 15, the non-usage of chemical adjuncts 7, 8, 12, 15, short duration of preoperative denosumab 7-9, and potential selection bias of patients 9, 12, 13.

Denosumab has been widely used in the treatment of GCTB after it was approved by the Food and Drug Administration 23. Recently, the use of denosumab as a neoadjuvant was attempted in the surgical therapy of GCTB that likely caused severe morbidity (e.g., Campanacci stage 3, severe soft tissue mass, joint resection, and spinal or sacral GCTB), and satisfactory results were obtained. *Rutkowski et al.* 6 investigated 222 GCTB patients who received preoperative denosumab; 48% (106/222) of patients were saved from surgery, and 38% (84/222) of patients had a less morbid surgery than originally planned. Although preoperative denosumab might benefit the surgical downstaging of GCTB, accumulating evidence has shown that the usage of preoperative denosumab might increase the risk of local recurrence after curettage 7-15. In our study, we found that GCTB treated with denosumab followed by curettage was significantly associated with a higher risk of local recurrence compared with curettage only (43% versus 20%, *P* < 0.01). The association between preoperative denosumab and local recurrence was further confirmed by most subgroup analyses (*P* < 0.05), except those with a small sample size (n < 59) (*P* = 0.09), sacral GCTB (*P* = 0.38), and usage of postoperative denosumab (*P* = 0.09). The high risk of local recurrence in patients treated with preoperative denosumab followed by curettage could be explained on the basis of existing evidence. Denosumab could cause irregular ossification within GCTB and form a rim of new bone that possibly contains neoplastic cells 22. During curettage, the complete removal of the ossification ring of new bone is difficult because of the unclear boundary between the tumor and normal tissues and osteosclerosis of ring. As a result, neoplastic cells remain in the rim of the new bone. Moreover, a translational study demonstrated that denosumab could eliminate only the giant cells in pathological tissues of GCTB, and the surviving stromal cells continue to proliferate *in vitro* after the withdrawal of denosumab 24. Therefore, the residual neoplastic cells in the ring of new bone might be the main cause of local recurrence after curettage. To overcome these drawbacks, some researchers advised the use of intraoperative C-arm fluoroscopy to distinguish the tumor boundary 7 and the application of ethanol with good penetrability to kill the residual neoplastic cells 25. However, selection bias may be another reason for the high local recurrence rate in the denosumab group because preoperative denosumab is usually used in complicated cases with a high local recurrence rate, such as patients with Campanacci stage 3, severe soft tissue mass, recurrent cases, and spinal or sacral GCTB 1.

Our findings showed that preoperative denosumab was not obviously associated with the increased risk of local recurrence in patients who received postoperative denosumab (P = 0.38). Postoperative denosumab might delay the local recurrence of GCTB by suppressing the activity of neoplastic cells in the ossification ring caused by preoperative denosumab 24. Therefore, the usage of postoperative denosumab is probably a feasible choice for delaying the local recurrence of GCTB; however, the optimal duration of postoperative denosumab remains unclear 26. Our findings also indicate that preoperative denosumab might not increase the risk of local recurrence of sacral GCTB treated with curettage (P = 0.42). Generally, sacral GCTB is difficult to surgically treat because of the surrounding sacral nerves; therefore, preoperative denosumab has been introduced to sacral GCTB in an attempt to decrease the operating difficulty and risk of intraoperative nerve injury 8, 15. However, our findings on sacral GCTB should be treated with caution because only two studies containing 37 patients were analyzed in this subgroup analysis 8, 15. Moreover, in *Chen et al*. 8, most patients (10/11) in the denosumab group received postoperative denosumab after curettage, which might delay the local recurrence of GCTB.

We have noticed that *Tsukamoto et al.* 27 performed a systematic review to explore the role of denosumab in the local recurrence of GCTB treated with curettage. However, several highlights in our study should be noted. First, we pooled the data from the included studies in the form of meta-analysis, which provided convincing evidence on clinical decision-making. Second, researchers focused on the effect of preoperative denosumab instead of postoperative denosumab on the local recurrence of GCTB. Therefore, to eliminate the influence of postoperative denosumab, we tried our best to extract the data of patients who received only preoperative denosumab in the denosumab group and also conducted the subgroup analysis based on the usage of postoperative denosumab. Third, we performed subgroup analysis in our study, which could offer comprehensive evidence on this topic. Fourth, we included two new studies in our analysis, which helped draw a more authoritative conclusion 8, 9.

Some limitations should be considered when interpreting our findings. First, all included studies had a retrospective design. As a result, selection bias of patients receiving preoperative denosumab might exist. To reduce the influence of this limitation, we performed subgroup analyses according to tumor site, Campanacci stage, and whether or not the patients received the previous surgery. Second, only nine studies containing 672 patients were included in the analysis, and the relatively small sample size might reduce the persuasiveness of our conclusion. The rarity of this disease might account for this limitation to some extent; multicenter studies should be carried out to deal with this limitation in the future. Third, the duration of preoperative denosumab might be a factor of local recurrence 14. However, we failed to find the optimal duration of preoperative denosumab because the data of individuals were unavailable. Fourth, many confounding factors, such as surgical technique, chemical adjunct, and invasion of adjacent tissues, were associated with the local recurrence of GCTG 1. A multivariate analysis should be conducted to determine whether preoperative denosumab is an independent risk factor of local recurrence in future work. Fifth, the follow-up period was relatively short in some included studies 8, 11, 15, especially in the denosumab group. As a result, the long-term effect of preoperative denosumab on the local recurrence of GCTB after curettage remains unclear. Sixth, although subgroup analyses were performed to detect the source of heterogeneity in the current study, heterogeneity was clear in specific subgroup analyses (e.g., sacral GCTB and usage of postoperative denosumab) and a random-effects model had to be used, which might have lowered the accuracy of the results.

## Conclusions

In conclusion, preoperative denosumab might increase the risk of local recurrence of GCTB treated with curettage. In consideration of our findings, preoperative denosumab should be used with caution in GCTB treated with curettage after the relevant benefits and risks have been balanced adequately. In our center, for patients receiving the curettage therapy, denosumab is tended to be used in patients with high risk of postoperative recurrence or potentially significant surgical morbidity, such as tumors with large soft-tissue extension, close to important neurovascular structures or joints of extremities, or large tumors located in the pelvis or spine. Generally, preoperative denosumab (120 mg) is subcutaneously injected on days 1, 8, and 15, with a loading dosage on day 28, and every four weeks, if required. Postoperative denosumab (120 mg) is subcutaneously injected monthly in 2 years after the surgery. However, in clinical practice, the duration of preoperative or postoperative denosumab varied a lot because of high expenses and patient's response to the drug 28. Therefore, multicenter randomized controlled trials should be conducted to further determine the effect of preoperative denosumab on the local recurrence of GCTB treated with curettage and explore the best duration of preoperative denosumab in the future.

## Supplementary Material

Supplementary table S1.

## Figures and Tables

**Table 1:** Characteristics of the included studies

Study	Country	Institution	Study design	Sample size (n)	Patients (LR/Total) (n)		Tumor site	Campanacci stage (I/II/III) (n)		Previous surgery
					Denosumab group	Control group		Denosumab group	Control group	
Agarwal et al. 2018 (7)	India	Single center	R	59	11/25	7/34	Pelvis, Sacrum, Extremity	0/8/17	0/9/25	Primary, Recurrence
Chen et al. 2018 (8)	China	Single center	R	21	3/11	3/10	Sacrum	0/0/11	0/0/10	Primary
Chinder et al. 2019 (9)	India	Single center	R	123	18/42	15/81	Pelvis, Extremities	0/25/17	9/56/16	Primary, Recurrence
Errani et al. 2018 (10)	Italy	Single center	R	247	15/25	36/222	Extremity	0/16/9	6/173/43	Primary, Recurrence
Fedenko et al. 2018 (11)	Russian	Multicenter study	R	20	6/16	2/4	Axial skeleton, Extremity	NR	NR	NR
Medellin et al. 2018 (12)	United Kingdom	Single center	R	59	4/4	17/55	Extremity	0/0/4	0/32/23	Primary
Scoccianti et al. 2018 (13)	Italy	Single center	R	21	5/12	1/9	Pelvis, Sacrum, Extremity	0/4/8	0/2/7	Primary
Urakawa et al. 2018 (14)	Japan	Multicenter study	R	106	6/31	15/75	Axial skeleton, Extremity	NR	NR	Primary, Recurrence
Yang et al. 2018 (15)	China	Single center	R	16	4/6	0/10	Sacrum	0/0/6	0/0/10	Primary

**Table 1A:** Characteristics of included studies.

Study	Chemical adjuncts	Duration of preoperative Denosumab (range) (month)	Postoperative Denosumab (n)	Follow-up time (range) (month)		Matched factors	Unmatched factors
				Denosumab group	Control group		
Agarwal et al. 2018 (7)	None	median 3 (1-13)	None	median 60 (27-90)	median 27 (12-42)	tumor site, tumor size, Campanacci stage, previous surgery	NR
Chen et al. 2018 (8)	None	median 2 (1-8)	10 patients	mean 18.3 (3-36)		Campanacci stage, tumor size	NR
Chinder et al. 2019 (9)	phenol and ethanol	mean 3 (1-7)	None	mean 32	mean 37	age, gender, symptom, history of trauma, pathological fracture, tumor site, tumor size, pulmonary metastasis, alkaline phosphatase, calcium, operation time, blood loss, complication	Campanacci stage
Errani et al. 2018 (10)	phenol	median 7 (6-12)	All patients	median 42.1, IQR 37.4-50.8	median 85.6, IQR 54.3-125.1	gender, Campanacci stage, previous surgery	age, tumor site, phenol
Fedenko et al. 2018 (11)	NR	mean 7	None	median 12.5		NR	NR
Medellin et al. 2018 (12)	None	mean 8.9 (3-19)	None	mean 75 (12-301)		NR	Campanacci stage
Scoccianti et al. 2018 (13)	cryotherapy	median 7 (4-7)	None	median 39 (14-55)	median 27 (18-92)	gender, tumor site, cement, bone graft, plate fixation	Campanacci stage
Urakawa et al. 2018 (14)	phenol, ethanol or liquid nitrogen	median dosage 6 (2-41)	10 patients	NR		NR	NR
Yang et al. 2018 (15)	None	median 4.5 (3-10)	None	mean 12 (7-18)	mean 35.3 (13-61)	age, gender, tumor site, tumor size, Campanacci stage	NR

R, retrospective; LR, local recurrence; IQR, interquartile range; NR, not reported.

**Table 2:** Risk of bias in the included studies by using the Newcastle-Ottawa scale

Study	Selection (⁕⁕⁕⁕)				Comparability (⁕⁕)	Outcome (⁕⁕⁕)			Overall
	Representativeness of exposed cohort	Selection of non-exposed cohort	Ascertainment of exposure	Outcome not present at start	Comparability on the basis of design or analysis	Assessment of outcome	Enough length of follow-up	Adequacy of follow up	
Agarwal et al. 2018	⁕	⁕	⁕	⁕	⁕⁕	⁕	⁕	⁕	9
Chen et al. 2018	⁕	⁕	⁕	⁕	⁕	⁕		⁕	7
Chinder et al. 2019	⁕	⁕	⁕	⁕	⁕	⁕	⁕	⁕	8
Errani et al. 2018	⁕		⁕	⁕	⁕⁕	⁕	⁕	⁕	8
Fedenko et al. 2018	⁕	⁕	⁕	⁕		⁕		⁕	6
Medellin et al. 2018	⁕	⁕	⁕	⁕		⁕	⁕	⁕	7
Scoccianti et al. 2018	⁕	⁕	⁕	⁕	⁕	⁕	⁕	⁕	8
Urakawa et al. 2018	⁕	⁕	⁕	⁕		⁕		⁕	6
Yang et al. 2018	⁕	⁕	⁕	⁕	⁕	⁕		⁕	7

**Table 3:** Subgroup analysis in the comparison of local recurrence between the denosumab and control groups

Variables	Included Studies	OR (95%CI)	P	I^2^ (%)	P for Heterogeneity	Model
**Ethnics**						
Asian	(7)(8)(9)(14)(15)	2.33 (1.40, 3.87)	<0.01^⁎^	46	0.11	Fixed
Caucasian	(10)(11)(12)	5.67 (2.70, 11.93)	<0.01^⁎^	42	0.16	Fixed
**Sample size (n)**						
< 59	(8)(11)(13)(15)	2.41 (0.88, 6.56)	0.09	49	0.12	Fixed
≥ 59	(7)(8)(10)(12)(14)	3.41 (1.51, 7.69)	<0.01^⁎^	61	0.04	Random
**Tumor site**						
Extremity	(10)(12)	8.83 (3.82, 20.40)	<0.01^⁎^	0	0.54	Fixed
Sacrum	(8)(15)	4.57 (0.11, 186.55)	0.42	75	0.05	Random
Both Axial skeleton and Extremity	(7)(9)(11)(13)(14)	2.18 (1.30, 3.65)	<0.01^⁎^	28	0.24	Fixed
**Campanacci stage**						
Matched	(7)(8)(10)(15)	4.28 (1.44, 12.73)	<0.01^⁎^	53	0.09	Random
Unmatched	(9)(12)(13)	4.23 (2.02, 8.85)	0.02^⁎^	0	0.49	Fixed
**Previous surgery**						
Primary	(8)(12)(13)(15)	4.96 (1.69, 14.49)	<0.01^⁎^	46	0.13	Fixed
Both Primary and Recurrence	(7)(9)(10)(14)	3.04 (1.32, 7.01)	0.02^⁎^	67	0.03	Random
**Chemical adjuncts**						
Yes	(9)(10)(13)(14)	3.26 (1.23, 8.59)	0.02^⁎^	67	0.03	Random
No	(7)(9)(12)(15)	3.81 (1.68, 8.66)	<0.01^⁎^	46	0.13	Fixed
**Preoperative Denosumab (month)**						
≤ 3	(7)(8)(9)	2.74 (1.46, 5.14)	0.02^⁎^	0	0.45	Fixed
> 3	(10)(11)(12)(13)(15)	6.41 (3.13, 13.13)	<0.01^⁎^	37	0.18	Fixed
**Postoperative Denosumab**						
Yes	(8)(10)(14)	2.05 (0.41, 10.18)	0.38	81	<0.01	Random
No	(7)(9)(11)(12)(13)(15)	3.69 (2.08, 6.55)	<0.01^⁎^	19	0.29	Fixed

OR, odd ratio; CI, confidence interval; ^⁎,^ P<0.05 indicating significant association between preoperative Denosumab and local recurrence.

## Abbreviations

GCTB: giant-cell tumor of bone
RANK: receptor activator of nuclear factor-kappa β
RANKL: RANK ligand
NOS: Newcastle-Ottawa scale
OR: odds ratio
CI: confidence interval
