# Supplementary material for: Preoperative Denosumab may increase the Risk of Local Recurrence of Giant-cell Tumor of Bone Treated with Curettage: A Systematic Review and Meta-analysis
Source: J Cancer. 2021 Jan 1;12(2):508–17. doi: 10.7150/jca.50575 (PMC7739008; doi:10.7150/jca.50575)
Supplement: Supplementary file 1 — Supplementary table S1. [file jcav12p0508s1.pdf]

Supplemental Table 1. Literature search strategy (search date: February 6th, 2020)

**PubMed**	**Query**	**Results**
#1	Search ((Giant Cell Tumor) OR Giant Cell Tumor of Bone) OR Osteoclastoma	13,737
#2	Search (((AMG162) OR Denosumab) OR Xgeva) OR Prolia	2,884
#3	#1 AND #2	**260**
**Cochrane database**		
#1	(Giant Cell Tumor of Bone):ti,ab,kw OR (Giant Cell Tumor):ti,ab,kw OR (Osteoclastoma):ti,ab,kw (Word variations have been searched)	130
#2	(Denosumab):ti,ab,kw OR (AMG162):ti,ab,kw OR (Xgeva):ti,ab,kw OR (Prolia):ti,ab,kw (Word variations have been searched)	937
#3	#1 AND #2	**11**
**Embase**		
#1	(Giant Cell Tumor or Giant Cell Tumor of Bone or Osteoclastoma).af.	8,216
#2	(Denosumab or AMG162 or Xgeva or Prolia).af.	8,755
#3	#1 AND #2	**463**
**Web of Science**		
#1	TS=(Giant Cell Tumor) OR TS=(Giant Cell Tumor of Bone) OR TS=(Osteoclastoma)	11,116
#2	TS=(AMG162) OR TS=(Denosumab) OR TS=(Xgeva) OR TS=(Prolia)	3,787
#3	#1 AND #2	**350**
